# Effect of biofilm formation on the antimicrobial activity of tigecycline against *Mycobacterium abscessus* in the hollow fiber infection model

**DOI:** 10.3389/fmicb.2026.1799565

**Published:** 2026-04-28

**Authors:** Hyunseo Park, Sara E. Maloney Norcross, Anthony J. Hickey, Mercedes Gonzalez-Juarrero, Bernd Meibohm

**Affiliations:** 1 Department of Pharmaceutical Sciences, College of Pharmacy, University of Tennessee Health Science Center, Memphis, TN, United States; 2 Engineering and Advanced Technology Department, RTI International, Durham, NC, United States; 3 Mycobacteria Research Laboratories, Department of Microbiology, Immunology and Pathology, Colorado State University, Fort Collins, CO, United States

**Keywords:** biofilm, hollow fiber infection model, *Mycobacterium abscessus*, non-tuberculous mycobacteria, PK/PD, tigecycline

## Abstract

**Introduction:**

Due to the inherent drug-resistance mechanisms and biofilm formation of *Mycobacterium* abscessus (*Mab*) that attenuate drug sensitivity, characterizing the impact of these factors on the pharmacological profile of antibiotics is critical to improve therapeutic outcomes.

**Methods:**

This study aimed to define the exposure-response relationship of tigecycline in *Mab* therapy and to simultaneously evaluate the effects of biofilm and resistance development on bacterial killing activity of tigecycline using a Transwell system and a Hollow Fiber Infection Model combined with pharmacokinetic/pharmacodynamic (PK/PD) modeling.

**Results:**

Dynamic time-kill assays conducted using the hollow fiber system, which mimicked tigecycline lung exposure under diverse intrapulmonary aerosol administration scenarios, demonstrated that high exposure to tigecycline effectively killed *Mab*. However, the pattern and timing of bacterial resistance development varied depending on the dosing regimen when exposure was insufficient for complete bacterial killing. Transwell-based *in vitro* tigecycline permeability study results revealed that the biofilm played a crucial role as a barrier to prevent molecular transfer of drug, eventually reducing the extent of exposure to *Mab* in biofilm by generating concentration gradients. The PK/PD model, integrating data from the *in vitro* dynamic time-kill assay and biofilm permeability study, adequately captured multiple factors, including dose-dependent bacterial killing, transition of *Mab* to less susceptible populations, biofilm formation, and biofilm-associated changes in permeability, all of which can influence the antibacterial activity of tigecycline.

**Discussion:**

A quantitative assessment of the impact of these factors modulating the bacterial pathophysiology provides insights into how *Mab* undermines the antibacterial efficacy of tigecycline, thereby ultimately contributing to the development of more efficacious tigecycline treatment strategies.

## Introduction

*Mycobacterium abscessus* (*Mab*) has emerged in recent decades as a significant pathogen responsible for pulmonary infections, particularly in the cystic fibrosis (CF) patient population (Recchia et al., 2023; Pearce et al., 2024). The pathological environment in CF lungs, characterized by excessively thick, sticky mucus (Gloag et al., 2021) and unique nutrient conditions, such as elevated heme that influences biofilm (BF) formation (Aftab et al., 2025) and increased glucose concentrations (Brennan et al., 2007), may provide a favorable environment for colonization and promote both drug tolerance and increased virulence of *Mab* (Hunt-Serracin et al., 2019). As a result, *Mab* pulmonary infection is now recognized as a major cause of morbidity and mortality in patients with CF (Wetzstein et al., 2022).

The optimal combination of antibiotic drugs, their dosing regimens, and their duration of therapy for the treatment of pulmonary *Mab* infection are not known. Nevertheless, macrolide-containing multidrug regimens that include at least three active drugs are generally recommended to overcome intrinsic multidrug resistance (Daley et al., 2020). Furthermore, the pathological property of *Mab* to form BF in lungs generates a physical barrier that restricts drug penetration. The barrier results in a concentration gradient within the BF, eventually leading to insufficient drug levels for effective killing of bacteria with reduced metabolic activity residing in the BF. Consequently, the persistence of subtherapeutic drug exposure may facilitate the development of acquired resistance. These complex factors are difficult to comprehensively evaluate using conventional *in vitro* or *in vivo* experimental systems (Nessar et al., 2012; Qvist et al., 2015; Fennelly et al., 2016; Luthra et al., 2018; Keefe and Bermudez, 2022) and are major translational barriers between preclinical studies and clinical outcomes, thereby contributing to the persistent challenge of developing effective therapeutic regimens against pulmonary *Mab* infection.

Intrinsic drug potency, typically measured as minimum inhibitory concentration (MIC) by *in vitro* susceptibility testing and interpreted alongside drug exposure metrics, such as area under the plasma concentration-time curve (AUC), maximum plasma concentration (C_*max*_), and exposure duration (Trivedi et al., 2013), is widely utilized to derive predictive parameters, including pharmacokinetic/pharmacodynamic (PK/PD) indices. These indices are often employed to inform clinical dosing strategies required to achieve appropriate antimicrobial efficacy against target pathogens (Vaddady et al., 2010). In the case of *Mab*, many clinical isolates appeared to be susceptible *in vitro* against various antibiotics (Wallace et al., 2002; Pearson et al., 2020; Portell-Buj et al., 2021). However, PK/PD index-based predictions have frequently failed to correlate with actual clinical outcomes, despite favorable MIC values obtained through standard assay systems (Portell-Buj et al., 2021; Pedersen et al., 2023). This discrepancy underscores the need for a more comprehensive evaluation of the factors influencing clinical outcomes in treating *Mab* infections.

In addition to intrinsic antimicrobial potency, patient-specific factors and pathogen-related mechanisms, such as BF formation and acquired resistance, may significantly modulate drug antibacterial activity *in vivo*. A quantitative understanding of BF formation and its role, particularly how and to what extent it impairs antibiotic activity, is of paramount importance for improving therapeutic efficacy. Furthermore, in-depth investigation into the mechanisms and conditions that drive the emergence of drug resistance is crucial for developing optimized treatment strategies, including dosing regimens and durations.

Tigecycline (TGC), a third generation glycylcycline antibiotic, has demonstrated remarkable clinical outcomes in more than 60% of CF patients with *Mab* infection (Wallace et al., 2014; Kwak et al., 2019), but is hampered by dose-limiting gastrointestinal toxicity that restricts its clinical utility in treating *Mab* infections (Kwon et al., 2019). Thus, targeted delivery of TGC to the lungs as site of *Mab* infection via inhaled administration has recently gained increasing interest (Maloney et al., 2023; Park et al., 2026b). Preclinical studies (Pearce et al., 2020) and a recently published clinical case report (Pedersen et al., 2023) support the therapeutic potential of inhaled TGC.

The Hollow Fiber Infection Model (HFIM) is a widely used *in vitro* tool that allows characterization of PK/PD relationships for antibiotics by mimicking the effect of dynamically alternating drug concentrations as observed in humans or in preclinical animal models and assessing their effect of bacterial growth and killing in the absence of any interfering immune system (Pieterman et al., 2021; Wale et al., 2024; Vignaud et al., 2025). While the effect of TGC on *Mab* in the HFIM has been investigated after simulated IV administration (Ferro et al., 2016), we utilized the HFIM to simulate pulmonary infection conditions and drug exposure following intrapulmonary aerosol (IPA) administration of TGC. During these experiments, bacterial growth, drug-induced bacterial killing, resistance development, and importantly, BF formation associated with *Mab* proliferation were monitored. To identify their relative contributions to overall therapeutic efficacy, a mathematical PK/PD model was developed to quantitatively describe the relationships among these elements. Model-based simulations were subsequently conducted to predict an optimal *in vivo* dosing regimen that can kill *Mab*, including less susceptible populations residing in BF. The predicted results were then compared with previously published *in vivo* efficacy data for IPA-administered TGC in murine infection models to evaluate model performance and potential utility in drug development and clinical practice (Pearce et al., 2020). These exploratory studies are expected to offer valuable insights for optimizing TGC therapy against *Mab*, and for informing broader treatment strategies involving other antibiotics targeting *Mab* or pathogens that impair therapeutic outcomes through BF formation during infection.

## Materials and methods

### Pharmacokinetics of TGC in mice after intrapulmonary aerosol administration

An *in vivo* pharmacokinetic and tissue distribution study with destructive sampling was performed following 10 mg/kg IPA administration in 8-week-old randomly mixed gender C57/BL6 mice (Charles River, Wilmington, MA). This strain has the same genetic background in which previously published efficacy studies were conducted (Pearce et al., 2020). For IPA administration, mice were administered with 50 μL of 4 mg/mL TGC (ChemShuttle cat #101150, Burlingame, CA) dissolved in saline solution using a FMJ-250 high-pressure syringe device (PennCentury, Philadelphia, PA, United States) with an attached MicroSprayer (MicroSprayer, model IA-C; PennCentury) as previously described (De Groote et al., 2018; Pearce et al., 2020). Animals (*n* = 3 per time point) were sacrificed at 0.083, 0.25, 0.5, 1, 3, 8, and 24 h post-IPA administration, and plasma and lung tissue were collected and stored at –80°C until quantification.

### Quantification of TGC in biological matrices

The lung samples were thawed and homogenized with four times their volume of PBS. A 50-μL aliquot of lung tissue homogenate or plasma sample was rigorously mixed with 200 μL of acetonitrile containing 0.1% formic acid for protein precipitation. After centrifugation, a 5-μL aliquot of the supernatant was injected into a LC-MS/MS triple quadruple mass spectrometer (AB Sciex, Foster City, CA) equipped with electrospray ionization in multiple reaction monitoring mode using the mass transitions of m/z 586.3 → 513.2 for TGC and m/z 595.5 → 514.3 for deuterated TGC (d9-TGC) used as an internal standard, respectively. The mobile phases consisted of water and acetonitrile/MeOH 75:25 (v/v), each containing 0.2 % FA. The analytes were eluted on a C18 column (4.6 × 50 mm, 3.5 μm, Waters, Milford, MA) in gradient mode. Pharmacokinetic parameters were calculated using standard non-compartmental analysis using a pooled sample approach and the software package Phoenix WinNonlin 8.0 (Certara, Princeton, NJ). Among these parameters, the *in vivo* terminal half-life (t_1/2_) in lung was used to determine experimental conditions of the *in vitro* dynamic time-kill assay in the HFIM.

### *In vitro* dynamic time-kill assay

**FIGURE 1:**
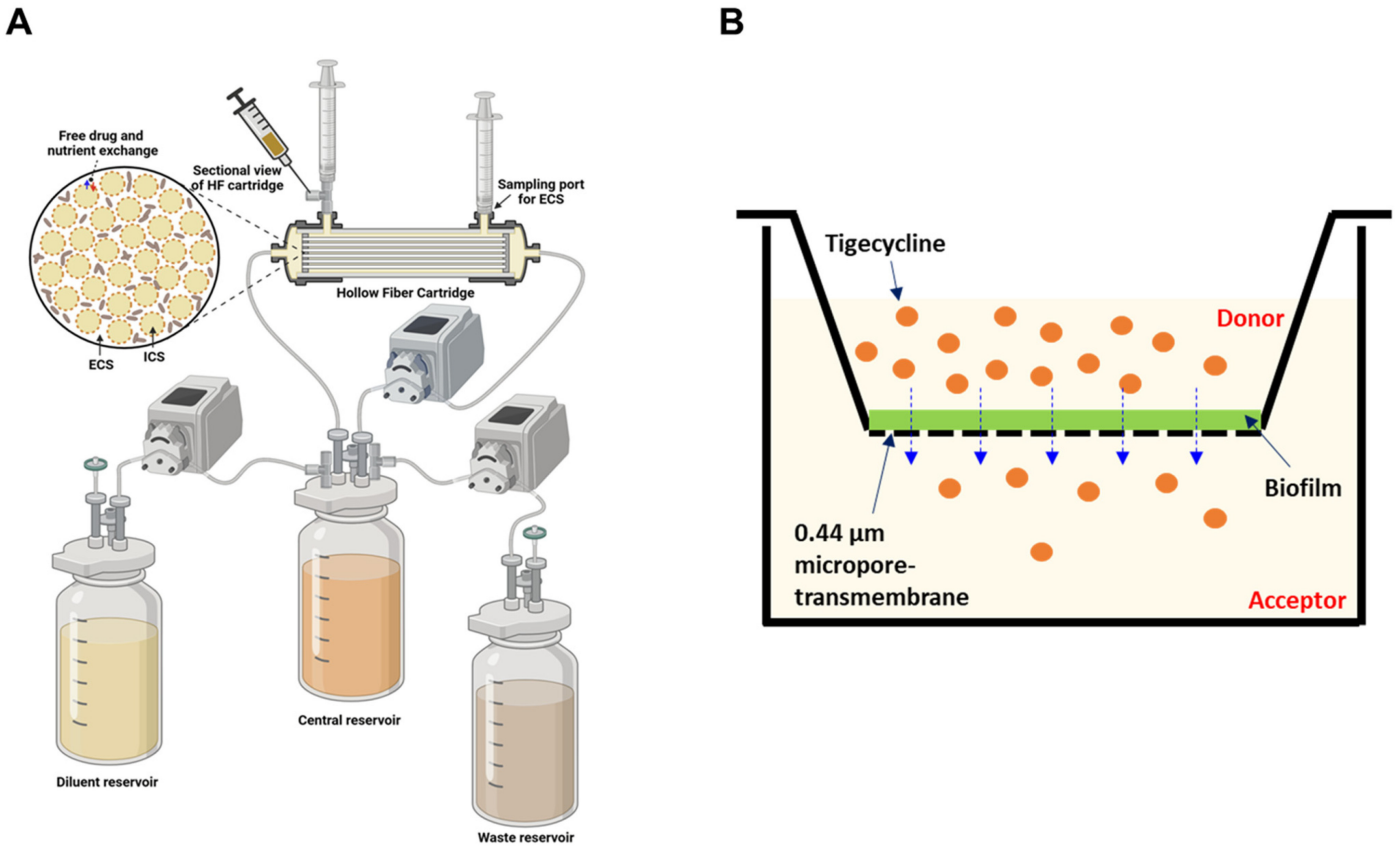
Schematic representation of applied *in vitro* model systems. **(A)** *In Vitro* Hollow Fiber Infection Model (HFIM) experimental system; and **(B)** Transwell™-based permeability assay platform. ECS extracellular capillary space; ICS intracellular capillary space.

To perform dynamic time-kill assay experiments under biologically meaningful conditions, drug exposure mimicking *in vivo* lung PK profiles obtained after IPA administration of TGC in mice was experimentally simulated in the HFIM (C3008, FiberCell Systems, Frederick, MD). The hollow-fiber cartridge used in these experiments was selected after screening available membrane types while considering the membrane characteristics and the hydrophilic nature of TGC, in order to identify the option that most accurately mimicked the desired pharmacokinetic profile. All studies described below were performed using Middlebrook 7H9 broth w/ OADC (Becton Dickinson, Sparks, MD) and incubated at 37°C. The flow rate of peristaltic pumps used for drug dilution of the HFIM system also considered the chemical instability of TGC in 7H9 as previously described (Park et al., 2026a), using Equation 1:


$$F\,l\,o\,w\,r\,a\,t\,e=\left(\frac{l\,n\,2}{t_{i\,n\,v\,i\,v\,o,1/2}}-\frac{l\,n\,2}{t_{7\,H\,9,1/2}}\right)\cdot V_{c\,e\,n\,t\,r\,a\,l}$$ (1)

where, t_*in vivo*,1/2_ and t_7H9,1/2_ represent the terminal half-life in mouse lung after IPA administration and the degradation half-life in 7H9 media reported previously in the literature (Park et al., 2026a), respectively, and V_*central*_ is the total volume of the central reservoir of the HFIM. The HFIM setup is illustrated in Figure 1A.

The *in vitro* PK of TGC in the peripheral and central compartment was assumed to be linear across the different dose levels since drug dilution using peristaltic pumps is the only mechanism associated with drug elimination from the system. Under multiple different exposure scenarios, the peripheral compartment of the system was inoculated with 15 mL of *Mab*, ATCC19977 (American Type Culture Collection, Manassas, VA), grown in 7H9 media under the early logarithmic phase at a concentration of 5 × 10^5^ colony forming units (CFU)/mL. Following a 3-h period for stabilization after *Mab* inoculation, TGC doses of 180, 600, 1,800, 6,000, and 18,000 μg were injected into the peripheral compartment of the HFIM for each group and repeated once daily for 7 days. The exposure resulting from these doses was equivalent to the unbound TGC concentrations after 3, 10, 30, 100, and 300 mg/kg TGC IPA doses in mice taking into account the protein binding properties of TGC in the 7H9 media as previously reported (Park et al., 2026a). Every 24 h from the beginning of the experiment until its conclusion, homogenous specimens of 100 μL of media were collected from the peripheral compartment of the HFIM. When BF formation was observed by visual inspection, two specimens were collected at each sampling time point from the peripheral compartment, one right before and one after a rigorous mixing step to distinguish BF-embedded *Mab* from planktonic *Mab*. These samples were then mixed with 900 μL of PBS and further serially diluted if needed. 100 μL of diluted samples were taken and plated on 7H10 agar plates (Becton Dickinson, Sparks, MD), and bacterial counts were enumerated after 3-day incubation at 37°C. All CFU assessments were conducted in triplicate.

### Drug susceptibility testing

If BF formation was visible, the two different specimens collected with and without BF also underwent susceptibility testing using the broth microdilution method with freshly prepared 7H9 to assess treatment-dependent changes in MIC. 100 μL of *Mab* culture at a concentration of 1 × 10^6^ CFU/mL, grown in 7H9, was mixed with an equal volume of media containing TGC serially diluted from 200 to 0.195 mg/L in a 96 well plate (Costar^®^, Corning, Glendale, AZ). The plate was incubated for 72 h at 37°C, followed by visual inspection, complemented by resazurin-based colorimetry (Sigma, St. Louis, MO). The MIC was determined as the lowest TGC concentration that prevented visible bacterial growth. Additionally, aliquots of the specimens containing BF were obtained from the terminal sample of the experiment and treated with TGC at concentration of 36 and 120 mg/L. These samples were transferred to a 96-well plate and monitored using a Cytation 5 Multi-Mode Reader (BioTek, Winnoski, VT) at 600 nm wavelength. The real time growth rates of *Mab* under the high TGC exposures were compared with naïve, drug unexposed bacteria as growth control.

### *In vitro* biofilm permeability measurements

To assess the impact of BF formation on TGC permeability, *Mab* was incubated on type-1 collagen coated Transwell membrane plates with 0.44 μm pore size (Corning Life Sciences, Kennebunk, ME) for 7 days to induce BF formation (Figure 1B). After incubation, the BF was washed twice with 500 μL of PBS and stained with 0.1% crystal violet (Sigma, St. Louis, MO) to confirm appropriate BF formation, ensuring it was sufficient for permeability assessments of TGC (Supplementary Figure 1; Ibaraki et al., 2020). For BF without staining, 0.5 and 2 mL of 7H9 was applied to donor and acceptor chambers, respectively, and 5 μL of TGC at 1 mg/mL was spiked into the donor side to initiate molecular transfer by diffusion toward the acceptor chamber. TGC transfer through the BF was measured by sampling the acceptor chamber at 0, 30, 60, 120, and 180 min after initiation. TGC concentrations in both acceptor and donor chambers were quantified using the LC-MS/MS method described above.

### Development of a mechanistic *in vitro* PKPD model

**FIGURE 2:**
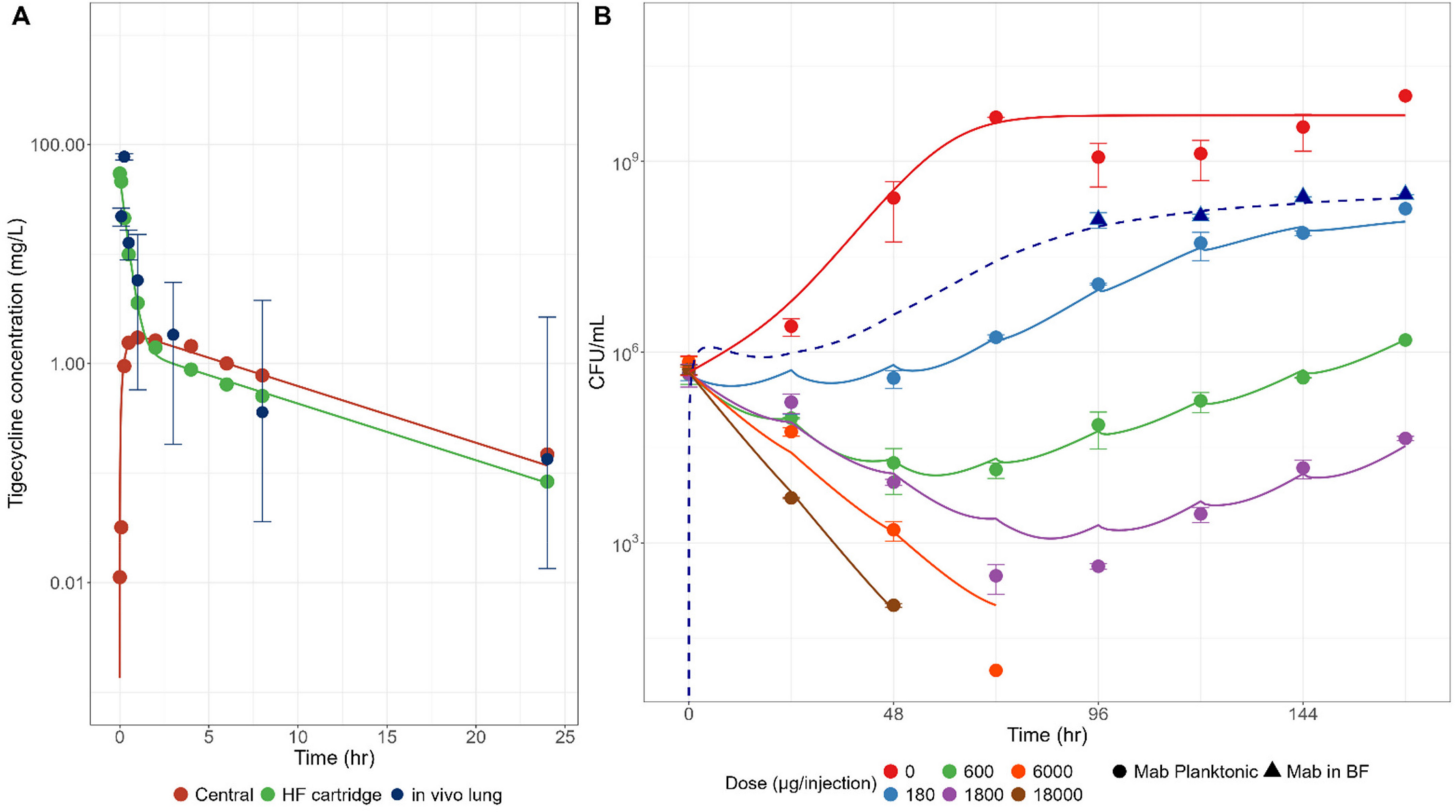
Bacterial killing of *Mab* in the hollow fiber infection model (HFIM). **(A)** Pharmacokinetic Comparison: The *in vivo* concentration-time profiles of TGC in mouse lungs following 10 mg/kg intrapulmonary aerosol (IPA) administration (mean ± SD) in comparison to the *in vitro* concentration-time profile simulated using the HFIM after a direct injection of 600 μg TGC into the peripheral compartment. **(B)** Time courses of bacterial counts in CFU/mL in the HFIM cartridge compartment assessed under the various TGC exposures achieved through once-daily direct injection of different TGC doses into the cartridge compartment for 7 days. Symbols and lines represent observed bacterial counts (mean ± SD) and model-based simulation profiles, respectively. BF-embedded *Mab* is indicated by a dashed line with triangle symbols. Each set of triplicate experimental results was individually captured by corresponding model-based simulation profiles.

**FIGURE 3:**
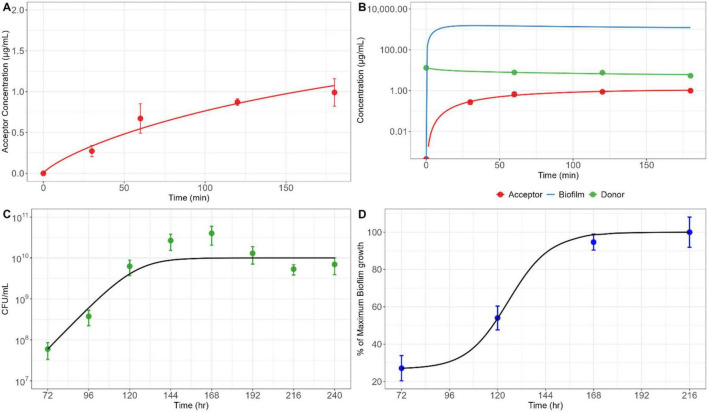
Biofilm (BF) permeability, formation and growth of embedded bacteria. **(A)** Experimentally measured TGC concentration changes in the acceptor chamber of the Transwell system with established BF, overlaid with Weibull function-based model predictions. **(B)** TGC concentrations-time courses in the acceptor and donor chambers, compared with those simulated within the BF using the PK/PD model. **(C)** Measured and modeled *Mab* growth within BF based on digitized data from Li et al. (2022) and **(D)** the BF formation under the corresponding experimental conditions. All symbols and solid lines represent in tripl\icate experimentally measured values (mean ± SD) and model-based simulation profiles, respectively.

Based on the generated bacterial count data and the simulated exposure profiles in the HFIM system, a PK/PD model was developed that integrated a two-compartment PK model component representing central and peripheral compartments of the HFIM with mechanistic components capturing bacterial growth, drug-induced bacterial killing, the emergence of intrinsic resistance (Figure 2), BF formation, and subsequent permeability changes (Figure 3). Using this approach, the model was designed to be expandable by incorporating experimental results obtained from different *Mab* strains, thereby allowing iterative updating and broader applicability of the framework. The model development described below and subsequent model-based simulations were performed using nonlinear mixed effect modeling in MonolixSuite*™* 2024R1 (Lixoft, Antony, France), with parameter estimation performed by the SAEM algorithm.

#### Biofilm formation

The BF formation was modeled as a function of BF maturity and overall density of *Mab* within the BF under corresponding experimental conditions, using two logistic functions (Equations 2 and 3). The dataset for BF formation modeling was digitized from the literature (Li et al., 2022):


$$\frac{d\,N}{d\,t}=N\cdot k_{0}\cdot \left(1-\frac{N}{N_{m\,a\,x}}\right)$$ (2)

$$\frac{d\,B\,F}{d\,t}=N\cdot k_{B\,F,f\,o\,r\,m}\cdot \left(1-\frac{B\,F}{B\,F_{m\,a\,x}}\right)-B\,F\cdot k_{B\,F,e\,l}$$ (3)

where N indicates the total colony forming unit (CFU) of *Mab* per volume (mL) in the BF matrix, including susceptible (*Mab-s*) and less susceptible *Mab* (*Mab-ls*) populations, and N_max_ indicates the maximum CFU of *Mab* per volume that can be maintained in the BF matrix. k_0_ is growth rate of *Mab* in BF. BF formation and its plateau phase were monitored using crystal violet staining and quantified by measuring the absorbance at 570 nm. Therefore, BF_max_, representing plateau of BF, was defined as 100%, and extent of BF formation at any given time was interpreted as the maturity of BF. The kinetics of BF formation was modeled with a first-order BF formation rate characterized by rate constant k_BF_,_form_ and a first-order BF elimination rate with rate constant k_BF_,_el_. The latter was fixed to a negligible value reflecting the sustained existence of BF once formed in the applied experimental system. The parameter estimate for K_BF_,_form_ derived at this step was integrated into the subsequently developed PK/PD model to comprehensively describe BF formation in the HFIM, under the assumption that the BF formation rates in the 24-well based system used to derive this value are comparable to those in the HFIM system.

#### Biofilm permeability

Time-dependent absorption changes of TGC into the BF were modeled using a Weibull function (Equation 4) for the time-dependent change of the first-order absorption rate constant k_a_,_BF_,


$$k_{a.B\,F}=\frac{\beta }{\alpha }\cdot {\left(\frac{t}{\alpha }\right)}^{\beta -1}$$ (4)

where k_a_,_BF_ is the first-order rate constant for the absorption of TGC from the media into the BF. α and β, estimated from experimental data, are the scale and shape parameter, respectively, and t is time. K_a_,_BF_ was further integrated into the PK/PD model to comprehensively describe TGC absorption into the BF in the HFIM, under the assumption that the permeability of TGC through the BF measured in the Transwell™ membrane system is representative of that in the HFIM system.

#### Pharmacokinetics

The pharmacokinetics of TGC in each compartment were described using the Equations 5–11 as below.


$$\frac{d\,A_{c\,e\,n\,t}}{d\,t}=A_{c\,a\,r\,t}\cdot k_{21}-A_{c\,e\,n\,t}\cdot k_{12}-A_{c\,e\,n\,t}\cdot k_{e\,l}$$ (5)

$$C_{c\,e\,n\,t}=A_{c\,e\,n\,t}/V_{c\,e\,n\,t}$$ (6)

$$\frac{d\,A_{c\,a\,r\,t}}{d\,t}=A_{c\,e\,n\,t}\cdot k_{12}-A_{c\,a\,r\,t}\cdot k_{21}-A_{c\,a\,r\,t}\cdot k_{a.B\,F}-A_{c\,a\,r\,t}\cdot k_{d\,e\,g}$$ (7)

$$C_{c\,a\,r\,t}=A_{c\,a\,r\,t}/V_{c\,a\,r\,t}=A_{c\,a\,r\,t}/\left(V_{C\,A\,R\,T}-V_{b\,f}\right)$$ (8)

$$\frac{d\,A_{b\,f}}{d\,t}=A_{c\,a\,r\,t}\cdot k_{a.B\,F}-A_{b\,f}\cdot k_{d\,e\,g}$$ (9)

$$C_{b\,f}=A_{b\,f}/V_{b\,f}$$ (10)

$$V_{b\,f}=V_{b\,f,m\,a\,x}\cdot \frac{B\,F}{B\,F_{m\,a\,x}}$$ (11)

where A_cent_, A_cart_ and A_bf_ represent TGC amount in the central compartment, the cartridge without BF compartment, and the BF compartment, respectively, C_cent_, C_cart_ and C_bf_ are the corresponding concentrations in these compartments, and V_cent_, V_cart_, and V_bf_ the corresponding volumes. V_CART_, the physical volume of the HFIM cartridge compartment, is the sum of V_cart_ and V_bf_. V_cart_ and V_bf_ gradually change depending on BF formation, determined by Equation 3. k_12_ and k_21_ are first-order rate constants for the transfer between central compartment and cartridge, k_el_ is the first-order elimination rate constant for the removal of drug from the central compartment as accomplished by exchange of media in the central compartment with fresh media, and k_deg_ is the first-order chemical degradation rate constant of TGC in 7H9 media at 37°C as previously determined (Park et al., 2026a).

#### Pharmacodynamics

As quorum sensing is believed to partially regulate bacterial growth, particularly in matured mycobacterial BF by regulating their metabolic activity (Solokhina et al., 2017), we assumed that the transition rate (K_tr_,_compartment_) of naïve *Mab* populations to less susceptible populations is governed by the net growth rate constant in the respective compartment (cartridge or BF), i.e., k_0,cart_ or k_0_,_BF_, which is influenced by the total population density of *Mab* in each compartment, i.e., the sum of the susceptible (N_s_) and less susceptible bacterial (N_ls_) subpopulations in each compartment relative to the maximal bacterial population that the compartment can accommodate (Equations 12 and 13). The transition was assumed to only occur when *Mab* is exposed to TGC regardless of residing location, i.e., media in the cartridge or BF.


$$k_{t\,r.c\,a\,r\,t}=k_{0,c\,a\,r\,t}\cdot \left(\frac{N_{s}^{c\,a\,r\,t}+N_{l\,s}^{c\,a\,r\,t}}{N_{m\,a\,x}^{c\,a\,r\,t}}\right)$$ (12)

$$k_{t\,r.B\,F}=k_{0.B\,F}\cdot \left(\frac{N_{s}^{B\,F}+N_{l\,s}^{B\,F}}{N_{m\,a\,x}^{B\,F}}\right)$$ (13)

Plausible differences in bacterial growth rate and their maximum population between BF and the media in the HFIM cartridge were described by adjustment terms, ADJ_Nmax_ and ADJ_k0_ for N_max_ and k_0_, respectively (Equations 14 and 15):


$$N_{m\,a\,x.B\,F}=N_{m\,a\,x,c\,a\,r\,t}\cdot A\,D\,J_{N_{m\,a\,x}}$$ (14)

$$k_{0.B\,F}=k_{0}\cdot A\,D\,J_{k_{0}}$$ (15)

For the antibiotic effect of TGC, it was assumed that TGC concentrations in media within the HFIM cartridge and the BF compartment were only responsible for bacterial killing in their respective compartments, and relocation of *Mab* between HFIM media and BF compartments was considered a reversible process (Lira et al., 2025). Differential Equations 16–19 describe population changes of *Mab* in the media within the HFIM cartridge and the BF. All parameters are differentiated by subscripts for susceptible and less-susceptible *Mab* populations. The first-order migration rate constants of *Mab* between the cartridge media and the BF are denoted by k_CtoB_ and k_BtoC_ for the transfer between cartridge media to BF and BF to cartridge media, respectively, and commonly applied to the N_s_ and N_ls_ populations. The antibiotic effect of TGC was modeled as a killing effect on N_s_ and N_ls_ in cartridge media and BF, respectively. This process assumed a common maximum kill rate constant K_kill_,_max_, differential sensitivities between sensitive and less sensitive bacteria reflected by KC_50_,_s_ and KC_50_,_ls_, respectively, and the respective TGC concentration in cartridge media and BF, C_cart_ and C_BF_, respectively. KC_50_,_s_ and KC_50_,_ls_ denote the TGC concentrations that elicit 50% of the maximum bacterial kill rate in the susceptible and the less susceptible population, respectively. KC_50_,_ls_ was expressed as the product of KC_50_,_s_ and the ratio of the MIC values for susceptible and less-susceptible *Mab* populations, MIC_s_ and MIC_ls_, respectively (Equation 20). Through this approach, the model was designed such that differences in susceptibility across bacterial populations, represented by MIC values, can influence the estimated potency of TGC, thereby allowing flexible extension of the modeling framework to other clinical isolates with different susceptibilities.


$$\frac{d\,N_{s}^{c\,a\,r\,t}}{d\,t}=N_{s}^{c\,a\,r\,t}\cdot \left(k_{0,c\,a\,r\,t}\cdot \left(1-\frac{N_{s}^{c\,a\,r\,t}+N_{l\,s}^{c\,a\,r\,t}}{N_{m\,a\,x,c\,a\,r\,t}}\right)-\frac{K_{k\,i\,l\,l,m\,a\,x}\cdot C_{c\,a\,r\,t}}{\left(K\,C_{50,s}+C_{c\,a\,r\,t}\right)}-k_{t\,r,c\,a\,r\,t}\right)-$$ (16)

$$N_{s}^{c\,a\,r\,t}\cdot k_{C\,t\,o\,B}+N_{s}^{B\,F}\cdot k_{B\,t\,o\,C}\cdot \frac{V_{b\,f}}{V_{c\,a\,r\,t}}\cdot$$

$$\frac{d\,N_{l\,s}^{c\,a\,r\,t}}{d\,t}=N_{s}^{c\,a\,r\,t}\cdot K_{t\,r,c\,a\,r\,t}+N_{l\,s}^{c\,a\,r\,t}\cdot \left(k_{0.c\,a\,r\,t}\cdot \left(1-\frac{N_{s}^{c\,a\,r\,t}+N_{l\,s}^{c\,a\,r\,t}}{N_{m\,a\,x,c\,a\,r\,t}}\right)-\frac{K_{k\,i\,l\,l,m\,a\,x}\cdot C_{c\,a\,r\,t}}{\left(K\,C_{50,l\,s}+C_{c\,a\,r\,t}\right)}\right)$$ (17)

$$-N_{l\,s}^{c\,a\,r\,t}\cdot k_{C\,t\,o\,B}+N_{l\,s}^{B\,F}\cdot k_{B\,t\,o\,C}\cdot \frac{V_{b\,f}}{V_{c\,a\,r\,t}})$$

$$\frac{d\,N_{s}^{B\,F}}{d\,t}=N_{s}^{B\,F}\cdot \left(k_{0,B\,F}\cdot \left(1-\frac{N_{s}^{B\,F}+N_{l\,s}^{B\,F}}{N_{m\,a\,x,B\,F}}\right)-\frac{K_{k\,i\,l\,l,m\,a\,x}\cdot C_{b\,f}}{\left(K\,C_{50,s}+C_{b\,f}\right)}-K_{t\,r,B\,F}\right)$$ (18)

$$-N_{s}^{B\,F}\cdot k_{B\,t\,o\,C}+N_{s}^{c\,a\,r\,t}\cdot k_{C\,t\,o\,B}\cdot \frac{V_{c\,a\,r\,t}}{V_{b\,f}}$$

$$\frac{d\,N_{l\,s}^{B\,F}}{d\,t}=N_{s}^{B\,F}\cdot K_{t\,r,B\,F}+N_{l\,s}^{B\,F}\cdot \left(k_{0.B\,F}\cdot \left(1-\frac{N_{s}^{B\,F}+N_{l\,s}^{B\,F}}{N_{m\,a\,x,B\,F}}\right)-\frac{K_{k\,i\,l\,l,m\,a\,x}\cdot C_{b\,f}}{\left(K\,C_{50,l\,s}+C_{b\,f}\right)}\right)$$ (19)

$$-N_{l\,s}^{B\,F}\cdot k_{B\,t\,o\,C}+N_{l\,s}^{c\,a\,r\,t}\cdot k_{C\,t\,o\,B}\cdot \frac{V_{c\,a\,r\,t}}{V_{b\,f}}$$

$$K\,C_{50,l\,s}=\frac{M\,I\,C_{l\,s}}{M\,I\,C_{s}}\cdot K\,C_{50,s}$$ (20)

Finally, *Mab* populations within the HFIM system, expressed in CFU/mL, were determined by Equations 21 – 23 and fitted to experimentally observed bacterial counts in the *in vitro* time-kill experiments.


$$N_{c\,a\,r\,t}^{T\,o\,t\,a\,l}=\left(N_{s}^{c\,a\,r\,t}+N_{l\,s}^{c\,a\,r\,t}\right)\cdot \left(\frac{V_{c\,a\,r\,t}}{V_{C\,A\,R\,T}}\right)$$ (21)

$$N_{B\,F}^{T\,o\,t\,a\,l}=\left(N_{s}^{B\,F}+N_{l\,s}^{B\,F}\right)\cdot \left(\frac{V_{b\,f}}{V_{C\,A\,R\,T}}\right)$$ (22)

$$N_{t\,o\,t\,a\,l}=N_{c\,a\,r\,t}^{T\,o\,t\,a\,l}+N_{B\,F}^{T\,o\,t\,a\,l}$$ (23)

For model qualification, diagnostic plots, such as observed versus population- or individual treatment group-predicted concentration, residual distribution, and relative standard error percentages (RSE%) for each parameter, were used to evaluate the model stability and robustness.

### Model-based Simulations

The final model was employed to simulate the antibacterial effect of multiple dosing regimens of TGC in the HFIM and to compare these simulations with the experimental results determined for doses found to be efficacious in *in vivo* GM-CSF KO mouse infection models, with the ultimate goal to evaluate predictability of the model (Pearce et al., 2020). Bacterial killing profiles for multiple *Mab* populations, including planktonic *Mab-s* and *Mab-ls* in the HFIM cartridge media and *Mab-s* and *Mab-ls* within the BF were simulated for different dosing regimens for 4 weeks of therapy with 1,000 replicates. The quantities used for efficacious dose-finding simulations included 3,000, 3,200, 3,400, and 3,600 μg/injection given once daily, and 3,200 μg/injection was considered the pulmonary exposure equivalent to a 60 mg/kg intrapulmonary aerosol dose in a mouse (20 g body weight). The results were plotted based on their relative susceptibilities (susceptible vs. less susceptible) and residing locations (cartridge media vs. BF). Among the simulated doses, the minimum effective dose (MED) was defined when the less sensitive population decreased over time in response to the TGC treatment. Using the same model, longitudinal changes in volume of BF and corresponding TGC concentration profiles within the BF were simulated. The simulation results were used to evaluate the impact of BF formation on effective TGC exposure in the BF and compared with the estimated half-maximal bacterial killing concentration for less sensitive *Mab*, KC_50_,_ls_. Lastly, to investigate the impact of pre-existing BF on antimicrobial activity of TGC after *Mab* infection, the bacterial killing effect of various TGC doses, including the simulated MED, was evaluated under delayed treatment scenarios when BF had time to establish.

### Global sensitivity analysis

The PK/PD model development was followed by a global sensitivity analysis (GSA), including Sobol and Partial Rank Correlation Coefficient (PRCC) sensitivity analysis. Satelli’s and Latin Hypercube Sampling (LHS) method were used to generate input sample matrices for the Sobol and PRCC method, respectively. The total number of parameters in the matrices were determined based on the number of parameters (p) and the number of initial sampling (*n* = 10,000), 2n⋅(p+1) (Zhang et al., 2015). The “mrgSolve” package in R (Metrum Research Group, Boston, MA) (Baron, 2024) was used for simulating core model output for all input combinations. The “sensobol” package in R was utilized to assess the relative impact of the individual model parameters involved in the PK/PD model on core model output, i.e. total bacterial burden (first-order indices, S_i_) as well as the impact of parameter interactions (total-order indices, T_i_) (Zhang et al., 2015; Puy et al., 2022). Additionally, the PRCC values were calculated using the “sensitivity” package in R to evaluate the relative importance along with longitudinal impact of each parameter on total bacterial burden. To interpret the results for both methods, the “ggplot2” in the R package was used to visualize the results (Wickham, 2009). All processes mentioned above were conducted using R version 4.3.3 (R Foundation for Statistical Computing, Vienna, Austria) (R Core Team, 2024) and RStudio 2023.12 (RStudio, Inc., Boston, MA) (RStudio Team, 2024).

## Results

### Pharmacokinetics of TGC and dynamic time-kill curves in the HFIM system

The pharmacokinetic profile of TGC *in vivo* was experimentally simulated using the HFIM, where the peripheral cartridge compartment represents drug disposition *in vivo* in the lungs. Following 600 μg of TGC injection into the peripheral compartment of the HFIM, the *in vitro* concentration-time profile of TGC closely aligned with the *in vivo* concentration-time profile observed in mouse lungs after 10 mg/kg IPA administration, during both the distribution and elimination phases. These results underscore that the applied media dilution was adequate to mimic the overall disposition of TGC *in vivo* in mouse lung post-IPA administration, thereby adequately accounting for the limited *in vitro* TGC stability in 7H9 media (Figure 2A; Park et al., 2026a).

Time-kill curves obtained at various dose levels ranging from 0 to 18,000 μg/injection (equivalent to 0–300 mg/kg given by IPA administration to a 20 g mouse) are presented in Figure 2B. As expected, bacterial growth in 7H9 media was rapid, reaching a plateau approximately 72 h post-inoculation. When TGC was injected to the peripheral compartment once daily for seven consecutive days with a 24-h interval, a dose-dependent bacterial killing effect was observed. At the lower dose groups, however, including 180, 600, and 1,800 μg/injection/day, bacterial regrowth occurred prior to termination of therapy, with longer delays with increasing dose levels. Complete bacterial killing was observed at the higher dose levels without regrowth within the observation period. For the lower dose levels, BF formation was observed at the bottom of the peripheral compartment in the lowest dose treatment group starting from day 4, when the bacterial population was sufficient to form BF. The number of BF-embedded *Mab* populations was higher than the planktonic *Mab* populations, consistent with previously reported results for the same *Mab* species, ATCC19977 (Li et al., 2022). Additional experiments were conducted to investigate whether there was a difference in drug susceptibility between the BF-embedded and the planktonic *Mab* populations.

### Identification of a less susceptible *Mab* population

From the results of the drug susceptibility testing performed with regrown *Mab* collected from media of the cartridge compartment, with or without BF contents, at the terminal observation point (Supplementary Figure 2), the MIC value determined for *Mab* populations containing BF was > 100 mg/L (Supplementary Figure 2, bottom), whereas those without BF were around 50 mg/L (Supplementary Figure 2, top). These values are significantly higher than the MIC of 3.13 mg/L for naïve *Mab* species without TGC exposure (Ferro et al., 2016; Pearce et al., 2020). When the bacterial populations containing BF were exposed to TGC at the final concentration of 120 and 36 mg/L and monitored at 600 nm wavelength, their growth rates were similar to the control group as well as the theoretical growth rate calculated based on normal growth of *Mab* in 7H9 media (Supplementary Figure 3). These observations indicate that the majority of *Mab* populations, regrown in the HFIM after initial decrease, exhibited significantly reduced susceptibility to TGC treatment.

### *In vitro* biofilm formation and permeability of TGC through biofilm

To evaluate the effect of BF formation on TGC distribution, we established a Transwell *in vitro* system to quantify the permeability of TGC in BF. The crystal violet staining method in this system confirmed that the transmembrane coated with type-1 collagen provided adequate conditions for *Mab* to generate a stable BF without leaking dye to the opposite side (Supplementary Figure 1). The permeation of TGC through BF on the Transwell membrane was characterized by a non-linear absorption rate that decreased over time. The time-dependent absorption was well described using the probability density function of a Weibull random variable (Figure 3A). The time-dependent absorption rate constant (K_a_,_BF_), expressed as probability density function of a Weibull random variable was adopted to describe absorption kinetics of TGC through the BF and integrated into the PK/PD model. When simulations of TGC absorption and its concentration profile within the BF were performed using a multi-compartment model with degradation rate constant of TGC (Supplementary Figure 4), rapid absorption into the BF was observed during the initial phase of absorption where the molecules of TGC started to transfer toward the BF, followed by gradual transfer to the acceptor chamber (Figure 3B). The BF played a significant role in impeding molecular transfer to the acceptor chamber, which created a concentration gradient of TGC that eventually reduced the drug reaching *Mab* within the BF. A mathematical model accounting for *Mab* growth within the BF and consequential BF formation (Equations 2 and 3) adequately captured the sigmoidal BF formation profile previously reported in the literature (Li et al., 2022), as shown in Figures 3C,D.

### Model-based mathematical integration of PK/PD and BF formation kinetics

**FIGURE 4:**
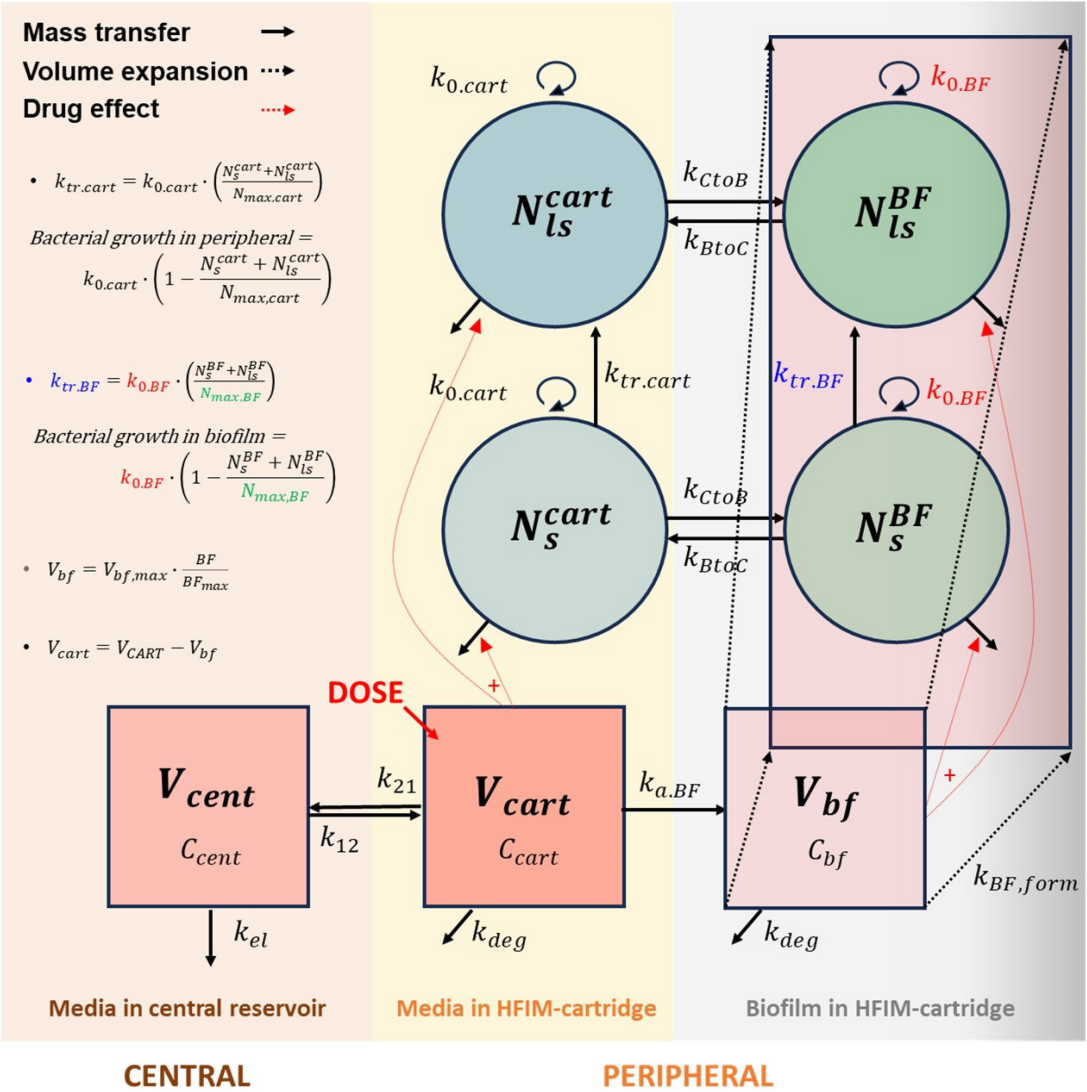
The final PK/PD model structure, accounting for (1) the pharmacokinetics and bacterial killing effect of TGC in each compartment, (2) the BF formation and its subsequent influence on *Mab* growth, including TGC permeability into BF and relocation of *Mab* between BF and HF-media, and (3) the bacterial transition from *Mab*-s to *Mab*-ls.

The final PK/PD model structure, which integrates the formation kinetics of the BF and the TGC permeability through the BF, is depicted in Figure 4. The integrated PK/PD model simultaneously described the PK of TGC in the cartridge compartment, which drives the bacterial killing effect of TGC, as well as the *Mab* killing and subsequent regrowth of the less susceptible populations in the HFIM cartridge and BF compartments (solid lines in Figure 4). Among the parameter estimates of the model summarized in Table 1, ADJ_Nmax_, an adjustment factor describing the difference in maximum bacterial load between planktonic and BF-embedded *Mab*, indicates that an approximately 4 times higher number of *Mab* can be residing within a volume unit of the BF matrix. On the contrary, ADJ_k0_, another adjustment factor accounting for a potential difference in net growth rate between planktonic and BF-embedded *Mab*, was estimated to be 0.96. Thus, the growth for BF-embedded *Mab* is nearly identical to the growth rate of planktonic *Mab*, being only 4% slower. The differences in maximum bacterial load and growth rate between planktonic and BF-embedded *Mab* may be due to multiple factors, such as quorum sensing and reduced nutrient accessibility (Lira et al., 2025).

**TABLE 1:** Parameter estimates of the integrated PK/PD model.

Model	Parameters	Unit	Descriptions	Estimate (median/RSE%)	Standard deviation of random effects (% CV/RSE%)
Pharmacokinetics	*V*_*cent*_	mL	Volume of central compartment of HFIM system	295.99/4.44	–
	*V*_*CART*_	mL	Volume of peripheral compartment of HFIM system\	11.63/6.59	–
	*k*_*12*_	1/h	The first-order transfer rate constant from central to peripheral compartment	0.08/9.16	–
	*k*_*21*_	1/h	The first-order transfer rate constant from peripheral to central compartment	3.05/5.29	–
	*k*_*el*_	1/h	The first-order elimination rate constant from the central compartment	0.12/4.87	–
	*k*_*deg*_	1/h	The first-order degradation rate constant of tigecycline	0.046/Fixed	–
Absorption through biofilm	α		Scale parameter for the Weibull distribution	518.12/Fixed	–
	β		Shape parameter for the Weibull distribution	0.78/Fixed	–
Biofilm formation	*V*_*BF*_	mL	The maximum volume of biofilm in HF cartridges	11.24/12.1	–
	*k*_*BF.form*_	%/CFU/h	Biofilm formation rate from *M. abscessus*	8.7 × 10^–10/^Fixed	–
Bacterial killing effect	*N_0_*	Log (CFU/mL)	The initial colony forming unit of *M. abscessus* in mL	5.68/13.2	–
	*K_0_*	1/h	The first-order net-bacterial growth rate	0.12/9.56	8.99/40.9
	*N*_*max*_	log (CFU/mL)	The maximum colony forming unit of *M. abscessus* in mL	9.43/81.5	10.04/24.0
	*K*_*kill.max*_	1/h	The first-order maximum bacterial killing rate constant for *M. abscessus*	0.26/4.99	9.60/35.5
	*KC*_50.*s*_	μg/mL	The half maximal inhibitory concentration of TGC against susceptible *M. abscessus* populations	0.23/29.8	–
	*k*_*CtoB*_	1/h	The first-order migration rate constant of *M. abscessus* from peripheral to biofilm	0.027/17.0	–
	*k*_*BtoC*_	1/h	The first-order migration rate constant of *M. abscessus* from biofilm to peripheral	0.00032/93.2	–
	*ADJ*_*N*_*max*__	Adjustment factor for maximum colony forming unit for biofilm-embedded *M. abscessus*	4.13/37.9	–
	*ADJ*_*k*_0__		Adjustment factor for bacterial growth rate of biofilm-embedded *M. abscessus*	0.96/4.11	–

The majority of the PK and PD parameters were estimated with acceptable precision. However, the parameters maximum number of *Mab* (N_max_), accounting for those bacteria residing in both peripheral and BF compartments, and the detachment rate of *Mab* from the BF (K_BtoC_) were not practically identifiable. Therefore, these estimates exhibited relatively higher RSE percentages compared to the other parameters. This is hypothesized to be due to the lack of sufficient information on the *Mab* populations growing in the BF compartment, as they were monitored over a limited time period and had likely not yet reached their true N_max_ in the lowest dose treatment group. Experimental evaluation of the detachment rate constant from BF to cartridge media could help address these identifiability issues. Nevertheless, none of the model diagnostic plots, including observed versus population- and individual-predicted concentration and residual distributions, exhibited skewed distributions, suggesting appropriate model performance (Supplementary Figure 5).

### Model-based simulations of minimum effective dose for bacterial killing

**FIGURE 5:**
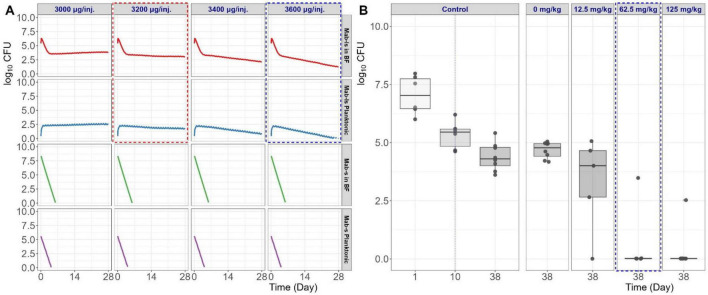
Minimum effective dose simulations for TGC against *Mab*. Final model-based bacterial killing profiles were predicted for various bacterial populations and their residing areas (each row in **A**), and the dose capable of gradual killing of *Mab*-ls was identified as the minimum effective dose (red dashed box in **A**). The 3,600 μg group, which demonstrated a distinct bacterial killing effect against *Mab*-ls (equivalent to 60 mg/kg IPA in mice *in vivo*, blue dotted box in **A**), accurately predicted the high efficacy observed *in vivo* in a murine infection model at a dose of 62.5 mg/kg in GM-CSF KO mice (blue dotted box in **B**). **(B)** Digitized from Pearce et al. (2020) and re-visualized for comparison purposes.

The bacterial killing effect of TGC against *Mab* populations with different metabolic state and microenvironment (i.e., planktonic vs. BF-embedded *Mab*) was simulated according to their relative susceptibilities (i.e. susceptible vs. less susceptible) (Figure 5A). Susceptible *Mab* populations, either suspended in cartridge media (N_cart_,_s_) or embedded in BF (N_BF_,_s_) were readily killed by TGC treatment. However, model-based simulation results for 4 weeks of dosing revealed that the bacterial growth of less susceptible *Mab* populations in both cartridge media (N_cart_,_s_) and BF (N_BF_,_ls_) declined only if more than 3,200 μg of TGC was injected into the cartridge compartment once daily. Considering that the *in vitro* TGC concentration-time profile in the cartridge compartment obtained following a 600 μg injection was comparable to the *in vivo* TGC lung exposure of a 10 mg/kg IPA dose in mice, the *in vivo* minimum effective dose of TGC, determined by the model-based simulations was approximately 53 mg/kg IPA administration in mice. The 3,600 μg dosage group, which exhibited a distinct bacterial killing effect, corresponds to approximately 60 mg/kg when converted to murine IPA administration. These prediction results aligned well with the results reported by Pearce et al. (2020), where a 62.5 mg/kg IPA dose of TGC administered in a 20 g mouse once daily, five times a week for 4 weeks, was highly effective *in vivo* in GM-CSF KO mice with *Mab* infections, resulting in bacterial killing below the quantification limit in 80% of the treated animals (Figure 5B). The comparison between our model-based simulations and the experimental findings *in vivo* underline the model’s predictive capabilities, supporting its utility for developing effective TGC dosing regimens.

### Evaluation of the impact of BF formation on TGC exposure and bacterial killing

**FIGURE 6:**
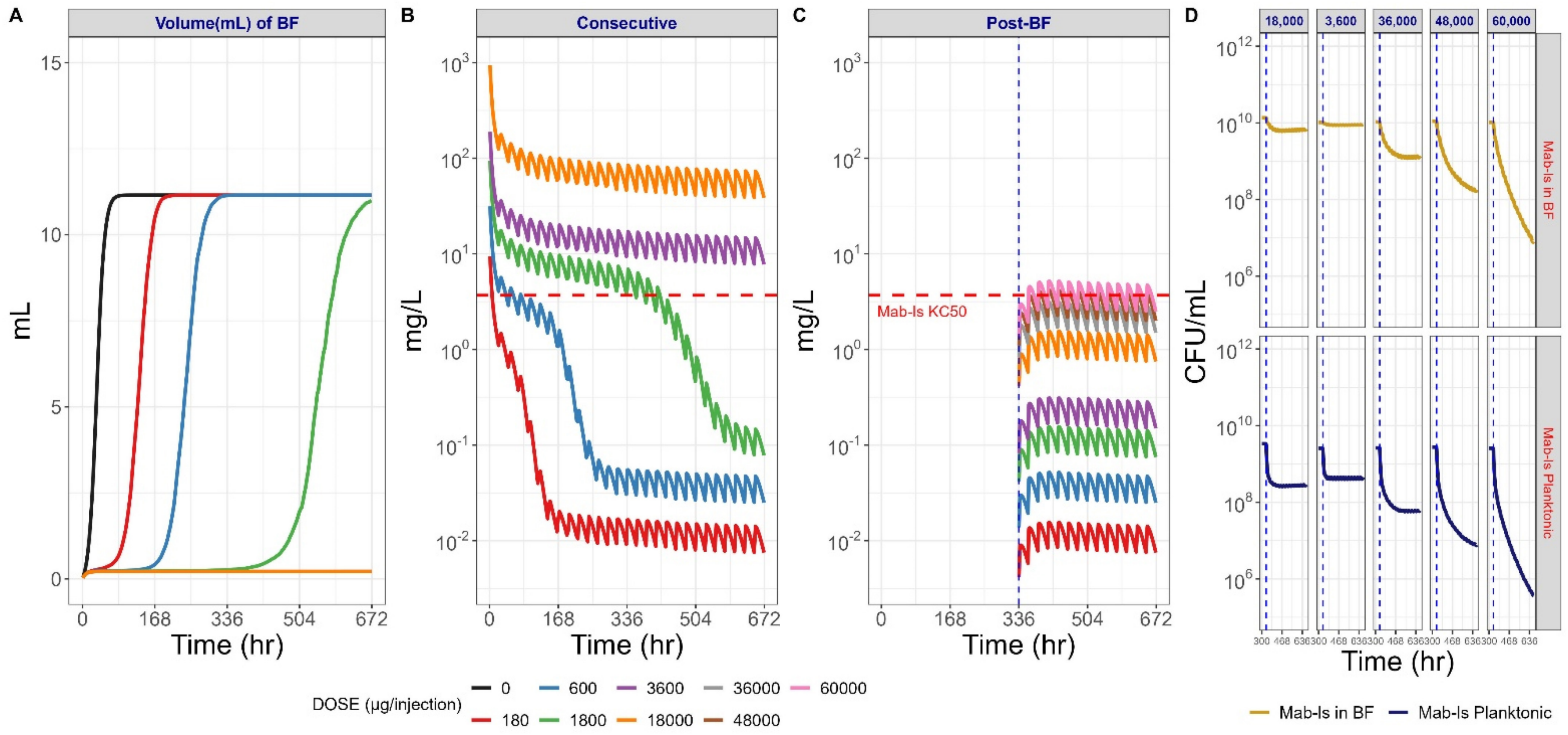
Effect of BF formation on TGC exposure and bacterial killing under various dose levels and treatment scenarios. **(A)** Longitudinal BF formation for different daily administered TGC doses simulated with the PK/PD model. **(B)** PK/PD-model simulated TGC concentration-time profiles within the BF compartment when once daily TGC dosing was initiated without delay following *Mab* inoculation. **(C)** PK/PD-model simulated TGC concentration-time profiles within the BF compartment when once daily TGC dosing was initiated once daily with 2-week delay following *Mab* inoculation, i.e., when a mature BF has developed. **(D)** Bacterial killing effect of high-dose TGC administered to the HFIM cartridge after a mature BF has been established. The dashed horizontal red lines in panels B and C indicate the half-maximal bacterial killing concentration for less susceptible populations of *Mab* (Mab-ls KC_50_).

Model-based simulation depicted in Figure 6A showed that BF formation depended on the daily dose of TGC which eventually determines the total number of bacteria capable of forming the BF. Higher daily doses resulted in lower numbers of bacteria in the HFIM system, less bacterial density, and thus less BF formation. In the same simulations, the timing and extent of BF formation appeared to significantly modulate the level of TGC penetration of the BF (Figure 6B), thereby reducing the TGC concentration below the KC_50_ for less sensitive *Mab*. This implies that BF formation has an important impact on limiting drug exposure to bacteria in the BF. This effect is particularly harmful to antibiotic therapy with TGC if the BF has been able to establish prior to initiation of antibiotic therapy. The simulation results in Figure 6C represent the changes in drug concentration within the BF under the assumption of delayed drug treatment post-*Mab* infection. In this scenario, the mature BF formed prior to drug administration significantly reduced TGC exposure within the BF compared to outside of the BF, thereby requiring a substantially greater dose of TGC to reach and sustain concentrations above the KC_50_ for *Mab*-ls. Unlike the time-kill curves in Figure 5A, the results indicate that an exceptionally high dose of TGC, more than 10 times higher, is required to eliminate both the intra-BF and extra-BF populations of less sensitive *Mab* in this scenario (Figure 6D).

### Global sensitivity analysis

In an effort to identify the most sensitive PK/PD model parameters and their underlying assumptions, variance-based Sobol indices were employed to identify influential parameters affecting the total bacterial burden in the cartridge compartment, the primary outcome variable of interest (Supplementary Figure 6A). The Sobol analysis indicated that parameters directly associated with both formation and function of BF, including the net absorption rate constant of TGC into the BF (k_a_,_bf_), the chemical degradation rate constant of TGC (k_deg_), bacterial relocation rate constant from cartridge to BF (k_CtoB_), and BF formation rate constant (k_BF_,_form_) significantly contributed to the total bacterial burden in the cartridge compartment in the 4-week treatment scenario, in addition to the intrinsic contributions from bacterial growth (k_0_ and N_max_) and drug effect (k_kill_,_max_ and KC_50_ for less sensitive *Mab*) parameters. PRCC was calculated at multiple time points for 4 weeks with 10-h intervals to explore potential time-dependent relationships (Supplementary Figure 6B). The overall results, including the rank order of parameter significance, were consistent with those of the Sobol method. However, the PRCC analysis additionally revealed that the contribution of each parameter to the total bacterial burden was relatively higher at the initial phase of treatment, which is presumably because the extent of initial bacterial killing influences the emergence of less susceptible populations, ultimately leading to an increased total bacterial burden.

## Discussion

In antibacterial pharmacotherapy, the bacterial burden, the establishment of a BF, and the impact of a BF on drug exposure are significant factors that may vary with the location and severity of a bacterial infection, and the associated bacterial strain. While there are several reports on the characteristics of BF produced by *Mab* and its influence on bacterial growth, its impact on pharmacologic activity of drugs is not well studied (Meliefste et al., 2024). However, considering the lipophilic composition and barrier-like structure of a BF (Belardinelli et al., 2021), it was anticipated that the drug exposure at the local infection site would change in case of BF formation, potentially leading to diminished antibacterial activity.

TGC has been described as an effective drug for treatment of *Mab* pulmonary infections (Wallace et al., 2014; Yang et al., 2022) and recommended as an option of multidrug regimens in treatment guideline (Daley et al., 2020). However, the exposure-response relationship for treating *Mab* infections with TGC, particularly under pathophysiological conditions, remains poorly understood. Recently, direct delivery of TGC to the lungs via IPA delivery or nebulization has been considered to maximize target exposure to TGC and resulted in favorable bacterial killing outcomes in rodents and humans (Pearce et al., 2020; Pedersen et al., 2023). This is likely because it ensures sufficient drug delivery to the target site, overcoming reduced drug sensitivity in some *Mab* populations and the additional access barrier caused by the BF under pathological conditions.

In this study, we utilized the HFIM to experimentally simulate TGC pharmacokinetics in lungs of mice following IPA administration and aimed to characterize (1) the exposure-response relationship for TGC with regard to bacterial killing of *Mab*, (2) the emergence of *Mab* populations with reduced sensitivity during the TGC treatment, and (3) BF formation under diverse TGC dose levels that can confer reduced TGC exposure at the infection site. We then employed a mathematical model to integrate these observations and experimental results in order to provide a quantitative assessment of the significance of each factor.

Ferro et al. (2016) reported that TGC demonstrated high efficacy against *Mab* pulmonary disease, achieving a one-log reduction in the HFIM when simulating an AUC corresponding to a 400 mg human dose. While this reduction is meaningful in terms of the proportion of bacteria eliminated by the drug, complete eradication of *Mab* was not observed, even at the highest treatment level simulating an 800 mg IV daily dosing in humans (Wallace et al., 2002). Moreover, the significance of this effect is further diminished when considering that the dose required to achieve this bacterial reduction is intolerable in humans based on the associated plasma concentration-related gastrointestinal adverse events (Rubino et al., 2012).

Following the direct injection of TGC to the HFIM cartridge in our experiments which mimicked multiple dose lung exposures in mice after IPA administration, a dose-dependent bacterial killing effect of TGC was observed alongside certain regrowth patterns which imply the emergence of less sensitive bacterial populations. These patterns appeared to be associated with the level of drug exposure which determined the extent of *Mab* killing prior to becoming less sensitive toward TGC. Therefore, understanding the required TGC exposure to prevent the emergence of less sensitive *Mab* populations and achieve effective bacterial killing is imperative for devising effective therapeutic regimens with TGC.

In a clinical case report on the treatment of pulmonary *Mab* infection using inhaled TGC, 25 mg twice daily successfully treated pulmonary *Mab* infection without relapse of *Mab* and other NTM infections during a 2-year follow-up period (Pedersen et al., 2023). Model-based simulations predicted that a TGC dose of 36,000 μg directly injected to the HFIM cartridge is required for bacterial killing in the mature BF (Figure 6D). Given that the volume of the HFIM cartridge is approximately 20 mL, the corresponding peak TGC concentration for the respective dose is then estimated to be around 1,800 μg/mL. In comparison, as the human epithelial lining fluid volume reported in previous literature ranges from approximately 15 to 70 mL (Rennard et al., 1986), the theoretically calculated initial peak concentration of TGC following inhalation of 25 mg TGC, assuming 100% delivery to the alveolar space post-inhalation, is estimated to range between approximately 350 and 1,600 μg/mL. Considering that the *Mab* strain isolated from the patient involved in the clinical case study exhibited resistance to TGC and other antibiotics *in vitro*, the dynamic time-kill assay applied in this study demonstrated reasonable predictive performance for effective TGC doses by accounting for the antibacterial effects against TGC-induced less susceptible *Mab* populations and covering clinically relevant exposure conditions. These findings highlight that the HFIM model, combined with model-based predictions, could serve as a valuable tool to design effective TGC treatment regimens and underlines the potential of translating the HFIM-generated *in vitro* time-kill data to clinical settings.

With regard to bacterial regrowth under low dose TGC therapy in the HFIM, one potential mechanism for the reduced sensitivity of *Mab* to TGC might be the expression of MAB_3542c, which encodes a protein homologous to RshA, the anti-sigma factor that negatively regulates SigH in *Mab*. Disruption of the RshA–SigH interaction under subinhibitory concentration leads to activation of MAB_3543c (SigH), thereby inducing a broad oxidative and envelope stress response that confers a less susceptible phenotype in *Mab* (Ng et al., 2018), each of which undergoes acute transcriptomic changes in both regions during the short-term incubation with subinhibitory concentrations of TGC for 24 h (Schildkraut et al., 2022). This would be in line with the regrowth of *Mab* observed 48 h after the first treatment in the HFIM experiment using 180 μg/injection, a dose which might be insufficient to kill the bacteria in the later phase of the dosing interval when TGC concentrations have substantially declined. Our current study, however, did not include genomic assessments and thus the outlined potential mechanisms are currently speculative and warrant further studies to elucidate this issue.

BF formation under this condition was observed from day 4 (96 h after the first treatment). Regrowth of *Mab* was gradually delayed with increasing daily dose levels but visible BF formation in the cartridge compartment was only observed in the 180 μg/injection experiment. Considering that the bacterial counts at the last day in the 600 μg/injection experiment were lower than those observed at day 4 in the 180 μg/injection experiment when the BF formation was visible, the threshold of local bacterial density required for visible BF formation is likely around 10^7^ CFU/mL. Taken together, our experimental observations and the literature observations (Epstein et al., 2004; Drlica and Zhao, 2007; Kaplan, 2011; Liu et al., 2024) emphasize that adequate control of bacterial burden in the initial phase of infections, by rapidly achieving therapeutically effective antibiotic concentrations, is crucial for high treatment success rates. This outcome would be promoted by reducing the likelihood of BF formation and emergence of less susceptible bacterial populations.

The results of our *in vitro* BF permeability study conducted using the Transwell system demonstrated a nonlinear molecular transfer of TGC through the BF to the acceptor chamber, which emphasized the need for proper characterization of the absorption kinetics of TGC into BF to elucidate its impact and correlation with other factors. The Weibull function, frequently used to describe nonlinear absorption processes, explained the absorption characteristics of TGC through the BF well (Figure 3A). In this context, BF also seemed to behave as a sequestration reservoir for the antibiotic, eventually decreasing the exact amount of TGC that can contact BF-embedded *Mab* (as indicated by the blue solid line in Figure 3B).

BF formation can affect overall bacterial growth because it significantly changes the bacterial microenvironment, including the population density of *Mab*, which may lead to differential bacterial growth kinetics (Chihara et al., 2015; Solokhina et al., 2017). A model parameter estimate, ADJ_k0_, describing the relative difference in bacterial growth rate in the BF matrix relative to media indicated an approximately 4% slower growth of BF-embedded *Mab* compared to planktonic-*Mab*. This observation may be attributed to several factors, including the more limited fluid flow in the BF matrix and the surface topography which may influence quorum-sensing dynamics (Mukherjee and Bassler, 2019). Through the parameter ADJ_Nmax_, the HFIM-based PK/PD model also effectively accounted for the relatively higher bacterial burden within the BF matrix, as corroborated by previous literature (Li et al., 2022). Both parameters together allowed for the quantitative PKPD-model based evaluation of bacterial killing against both planktonic and BF-embedded *Mab* populations.

Conventional planktonic-*Mab*-based *in vitro* susceptibility testing does not consider the protective effect of BF for embedded *Mab*. Previous reports suggested that the minimum concentrations required to kill BF-embedded bacteria can be up to 1,000-fold higher than the MIC of antibiotics against planktonic bacteria (Olson et al., 2002), which aligned well with our *in vitro* susceptibility test results (Supplementary Figure 2) and persistent growth of *Mab* under high TGC exposure (Supplementary Figure 3). As shown in model-based simulation for the BF formation and corresponding changes in TGC concentration within the BF compartment depicted in Figure 6, TGC concentrations in the BF were observed to be gradually declining due to the BF-mediated time-dependent delayed absorption (Figures 6A,B). Furthermore, the model-based simulations assessing the impact of post-BF formation revealed that fully mature BF does more significantly hinder the bacterial killing effect of TGC and requires an approximately 10-times higher dose to kill *Mab* (Figures 6C,D). These findings suggest that in cases of chronic infections resulting from inadequate treatment where BF has been established at the infection site, achieving therapeutic effects may require substantially higher dosing regimens or more intensive therapeutic strategies.

The HFIM is a well-established *in vitro* tool to assess dynamic PK/PD relationships for antibiotics in the absence of interfering immune system effects, that has been widely used in optimizing the anti-infective pharmacotherapy (Sadouki et al., 2021). Its application includes the evaluation of numerous antimicrobial agents against *Mab* (Ferro et al., 2016; Gibson et al., 2024; Vignaud et al., 2025). The experimental work presented in this manuscript builds on this previous work and adds three novel aspects: Instead of simulating drug concentrations in plasma or blood as usually relevant for systemically administered anti-infective therapy, it focuses on simulating antibiotic concentrations in lung tissue after local drug delivery by intrapulmonary aerosol administration. In addition, serial sampling of microorganisms during therapy is not only performed from planktonic organisms, but also for bacteria embedded in the BF formed in the hollow fiber compartment. This dual sampling strategy is novel and facilitates the simultaneous characterization of TGC activity against both, planktonic and biofilm embedded *Mab*. Furthermore, integration of the permeability limitations for TGC in the BF as assessed by the Transwell experiments into the PK/PD modeling approach allowed delineation of intrinsic sensitivity differences between these two bacterial populations from BF-related drug access limitations.

To our knowledge this is the first presentation of model-based assessments that quantitatively explain the critical role that BF formation plays as a defense mechanism for *Mab*. Therefore, it is essential to contemplate strategies to mitigate BF-related therapeutic vulnerabilities. Potential approaches may include (1) development of BF formation inhibitors that loosen the BF matrix, thereby enhancing permeability of antibiotics to gain access to BF-embedded *Mab*, (2) proper use of symptom-relieving agents that can reduce the aggregation of *Mab* suspended within the abnormal sticky mucus, especially in CF patients, and importantly, (3) finding accurate ways of direct drug delivery to the target bacteria in the lung, such as inhalation of antibiotics, and their proper dosing regimens in clinical conditions (Henke and Ratjen, 2007; Li et al., 2022; Volpi et al., 2022; Pedersen et al., 2023).

The current study demonstrated that the HFIM can be effectively utilized to quantitatively evaluate BF formation and its associated impact while also highlighting the feasibility of interpreting these dynamic processes through a model-based analysis approach. Although the current PK/PD-model analysis improved our understanding of the effect of BF on the therapeutic activity of antimicrobials, the approach still has several limitations: The BF formation kinetics were assessed and parameterized in an *in vitro* system using a lab strain of *Mab* (ATCC19977). Therefore, to achieve improved clinical relevance, it is essential to comprehensively consider not only the differing susceptibilities observed in clinical isolates but also factors such as BF formation rates that may vary under real physiological conditions, as well as functional changes of BF arising from multiple biological factors, including complex interactions with immune cells.

An additional limitation of the model is related to the fact that the growth characteristics of the less susceptible *Mab* populations were estimated from a limited set of growth curve data rather than measured directly. In principle, this limitation could be addressed if the different bacterial subpopulations could be experimentally separated and their growth rates independently quantified, which would be technically challenging.

Furthermore, the HFIM experiments in this manuscript were performed in 7H9 media. While this facilitates interpretability and compatibility of the results to previously performed HFIM studies with *Mab*, simulated lung fluid may have been more similar to the expected environment of interaction between *Mab* and TGC after pulmonary aerosol administration. Thus, improved quantitative assessments of the impact of factors modulating the pathophysiological properties of *Mab* are expected to further enhance our understanding of how BF undermines bacterial killing as well as of what TGC concentrations are required to completely kill *Mab* at the infection site, including less susceptible populations.

## Data Availability

The raw data supporting the conclusions of this article will be made available by the authors upon request, without undue reservation.
