# Supplementary material for: Effect of biofilm formation on the antimicrobial activity of tigecycline against Mycobacterium abscessus in the hollow fiber infection model
Source: Front Microbiol. 2026 Apr 28;17:1799565. doi: 10.3389/fmicb.2026.1799565 (PMC13161170; doi:10.3389/fmicb.2026.1799565)
Supplement: Supplementary file 1 [file Data_Sheet_1.pdf]

Supplementary Material

To

Effect of Biofilm Formation on the Antimicrobial Activity of Tigecycline against Mycobacterium abscessus in the Hollow Fiber Infection Model

Hyunseo Park, Sara E. Maloney Norcross, Anthony J. Hickey,

Mercedes Gonzalez-Juarrero, Bernd Meibohm

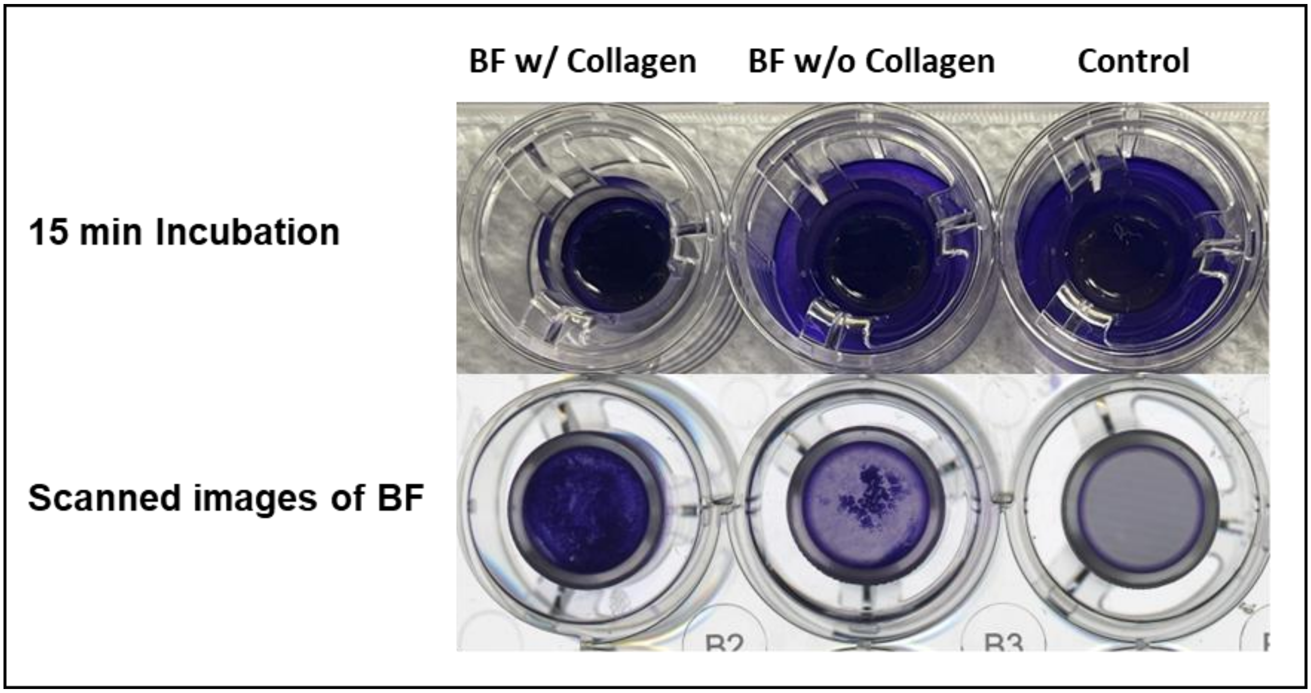

Figure S1. **Biofilm (BF) formation in the Transwell^{TM} system**. BF formed on the Transwell^{TM} membranes coated with or without type-1 collagen was stained using 0.1% crystal violet. The BF grown under conditions without collagen was inadequate for proper BF formation, as crystal violet dye permeated toward the acceptor chamber within 15 minutes.

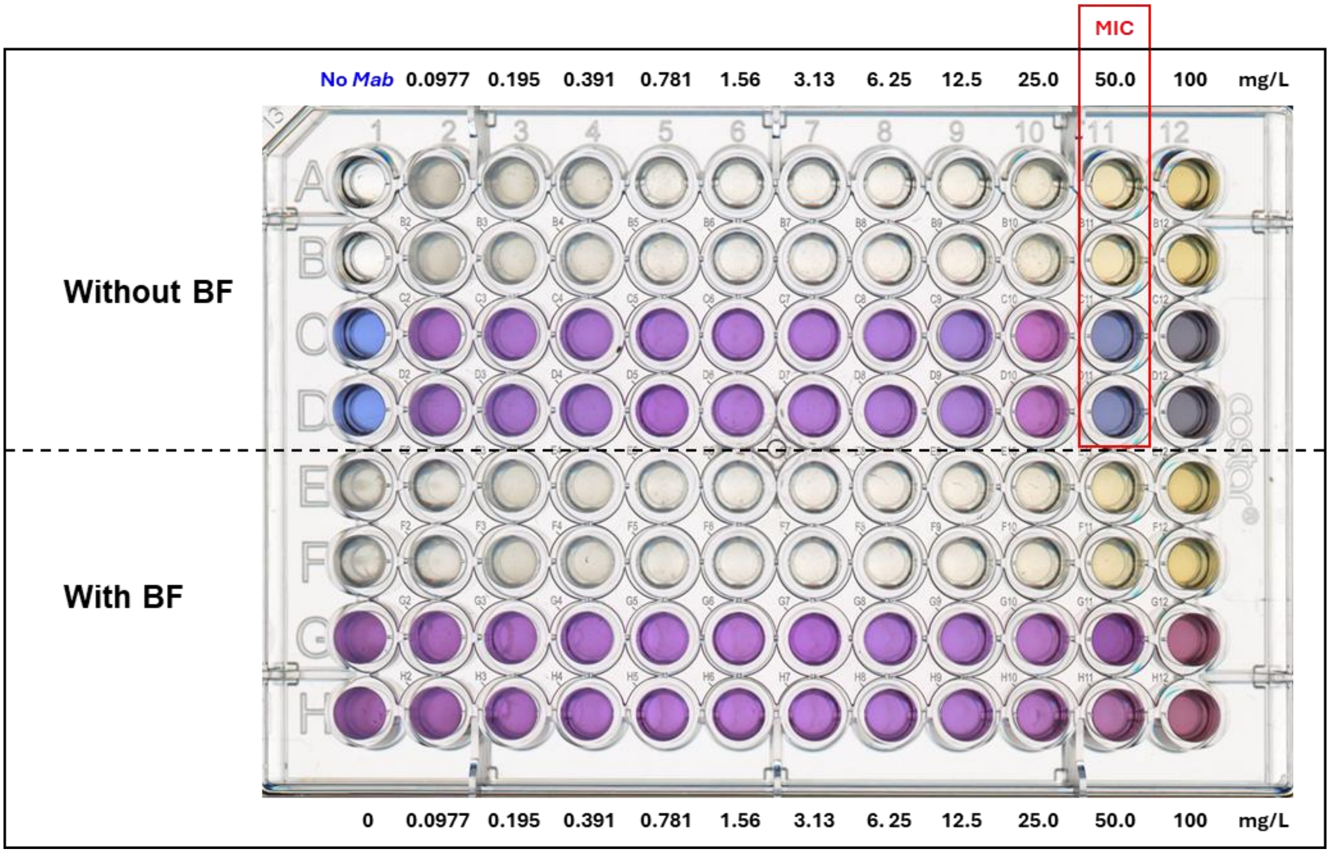

Figure S2. **Susceptibility testing of *Mab* isolated from the Hollow Fiber Infection Model (HFIM)**. Results of *in vitro* tigecycline susceptibility testing against *Mab* isolated from the *in vitro* time-kill assay in HFIM, with biofilm (BF) (lower half) or without BF (upper half). The determined MIC is indicated within the solid red box. MIC values for *Mab* incubation with BF exceeded the highest tested concentration.

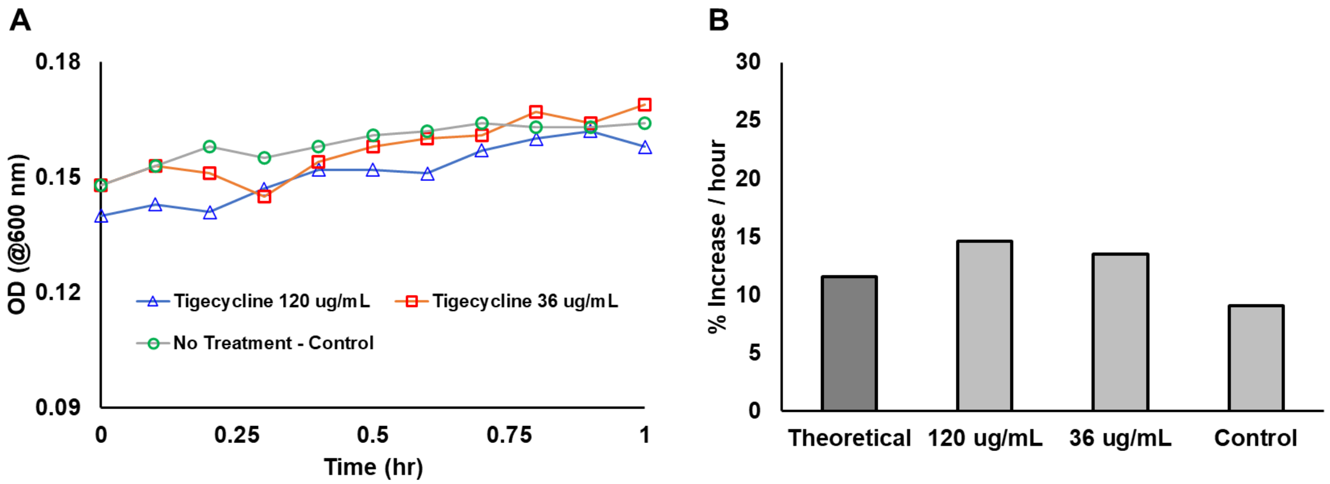

Figure S3. **Assessment of growth rates for less susceptible *Mab* populations**. Real-time growth of less susceptible *Mab* populations isolated from the *in vitro* time-kill assay in the HFIM. Bacterial growth was monitored as optical density at 600 nm wavelength using a spectrophotometer, with or without tigecycline treatment (A). The percentage increase after incubation was compared to the theoretical growth of *Mab* in 7H9 media (B).

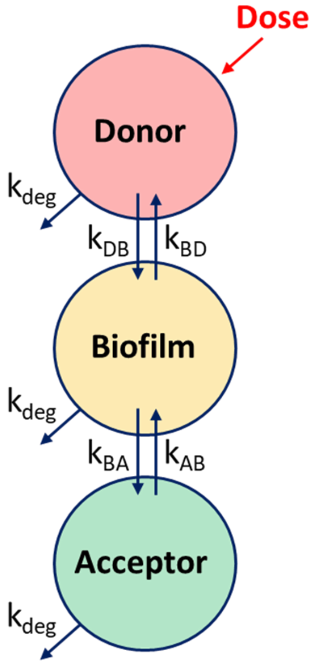

$$\frac{dA_{Donor}}{dt} = -A_{Donor} \cdot K_{DB} + A_{Biofilm} \cdot K_{BD} - A_{Donor} \cdot K_{deg}$$

$$\frac{dA_{Biofilm}}{dt} = A_{Donor} \cdot K_{DB} + A_{Acceptor} \cdot K_{AB} - A_{Biofilm} \cdot K_{BD} - A_{Biofilm} \cdot K_{BA} - A_{Biofilm} \cdot K_{deg}$$

$$\frac{dA_{Acceptor}}{dt} = A_{Biofilm} \cdot K_{BA} - A_{Acceptor} \cdot K_{AB} - A_{Acceptor} \cdot K_{deg}$$

$$C_{Donor} = A_{Donor}/(V_{Donor} - V_{Biofilm})$$

$$C_{Biofilm} = A_{Biofilm}/V_{Biofilm}$$

$$C_{Donor} = A_{Acceptor}/V_{Accpeptor}$$

Figure S4. **Modeling of tigecycline uptake into the biofilm (BF)**. Three-compartment model structure (left) and corresponding differential equations (right) were used to describe the absorption of tigecycline into the BF and simulate its concentration profile in BF as a function of time.

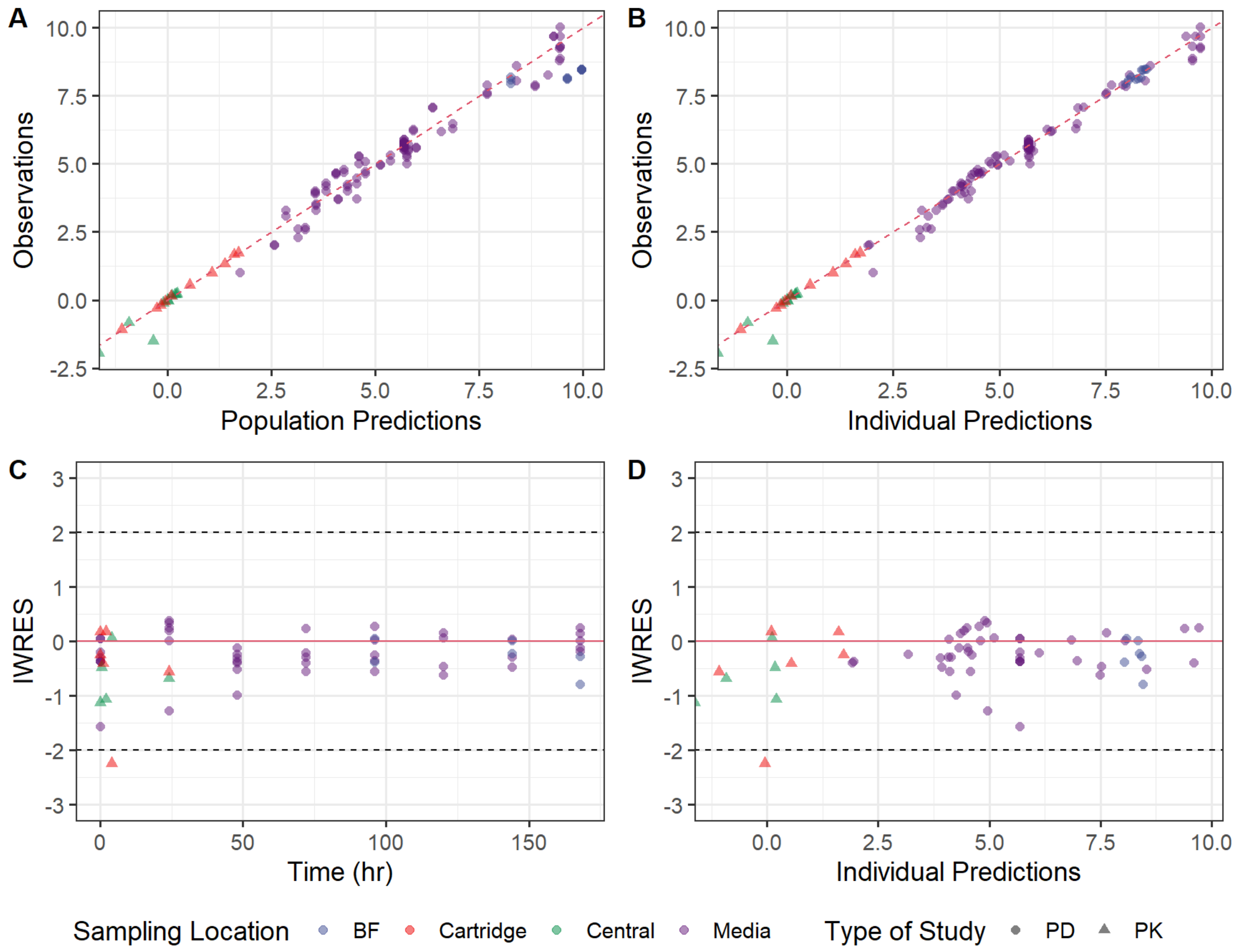

Figure S5. **Model diagnostic plots.** Observed versus population- (A) or individual-(B) predicted concentration, residual distribution versus time (C), and residual predicted versus predicted concentrations (D). IWES Individual weighted residual.

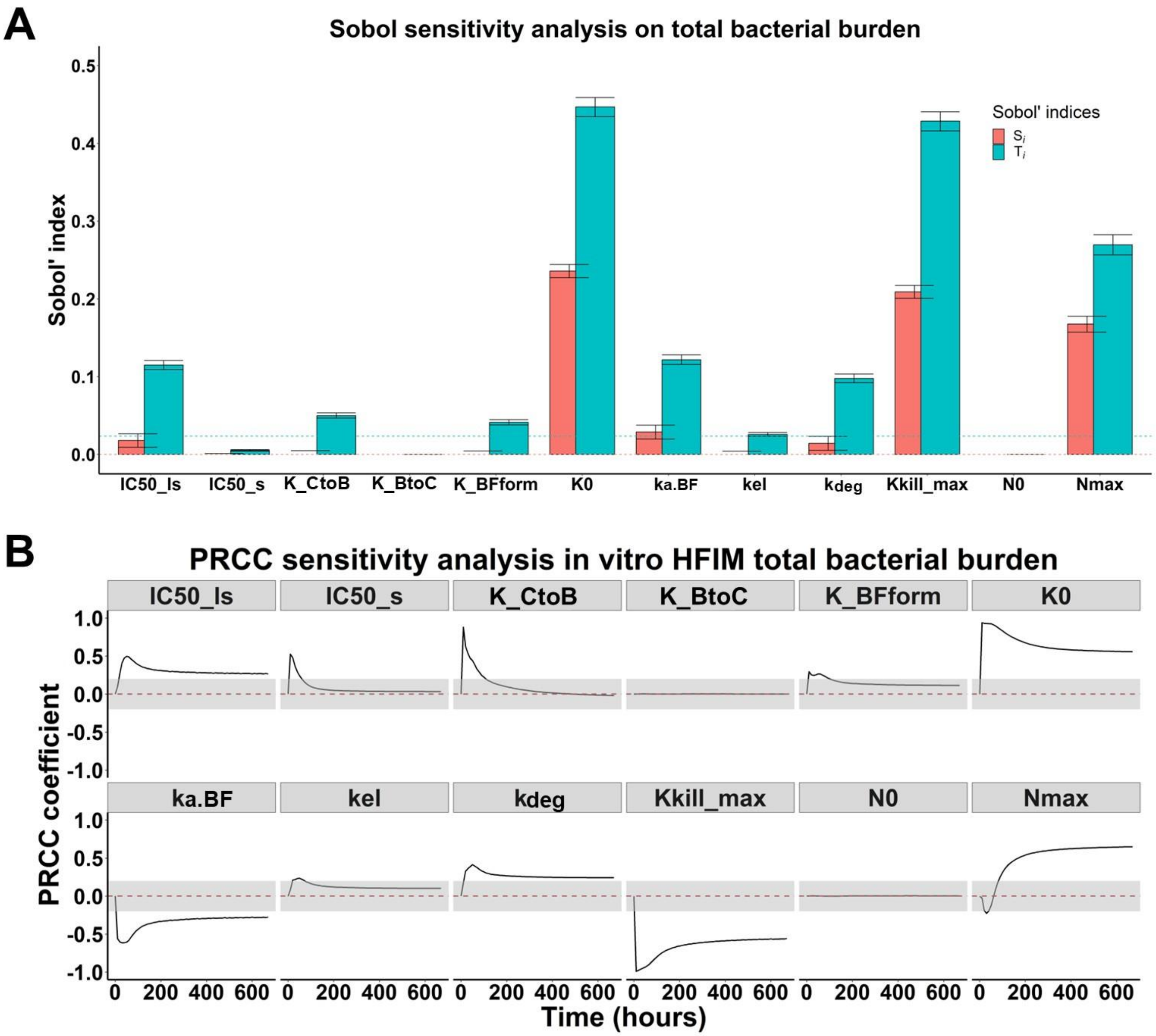

Figure S6. **Global sensitivity analysis on total bacterial burden using the Sobol (A) and the PRCC (B) method.** Vermilion and mint colors in panel A represent the first and total order Sobol' indices, respectively, with error bars indicating the 95% confidence interval for each index. The horizontal dashed lines denote the upper limit of the T_i and S_i indices of the dummy parameter, identifying parameters contribution to total bacterial burden. The shaded area in gray in panel B is the upper and lower 20% interval determining significant contribution of each parameter.
